# Venous sinus stenting improves cerebral autoregulation in a patient with venous sinus stenosis: a case report

**DOI:** 10.1186/s12883-019-1595-9

**Published:** 2020-01-08

**Authors:** Meiyan Jia, Zhen-Ni Guo, Hang Jin, Xiuli Yan, Mingchao Shi, Xin Sun, Hongyin Ma, Shan Lv, Yi Yang

**Affiliations:** 1 Department of Neurology, The First Hospital of Jilin University, Changchun, China; 2 Department of Neurology, Clinical Trial and Research Center for Stroke, The First Hospital of Jilin University, Changchun, China

**Keywords:** Venous sinus stenosis, Cerebral autoregulation, Venous sinus stenting, Cerebral circulatory, Case report

## Abstract

**Background:**

Venous sinus stenosis (VSS) is a type of cerebral venous vascular disease. Cerebral autoregulation is an indicator of cerebral arterial function. The cerebral circulatory system is composed of the venous system and arterial system. Impaired venous function may affect arterial function. Thus, cerebral venous stenosis may influence cerebral autoregulation.

**Case presentation:**

In this case, a 50-year-old woman with transient blindness and headache was admitted to the hospital. The patient was diagnosed with VSS. A stent was placed at the stenosis. The stent released the intravenous pressure and remitted the patient’s symptoms. Measurements of dynamic cerebral autoregulation (dCA) were performed at 3 time points: before stenting, after stenting, and 3 months later. The dCA gradually improved after stenting.

**Conclusion:**

VSS may have an influence on cerebral autoregulation, and effective treatment improves cerebral autoregulation in patients with VSS.

## Background

Cerebral autoregulation (CA) is a physiological mechanism that maintains a stable cerebral blood flow within a wide range of blood pressure fluctuations. CA is an indicator of cerebral arterial function. Research has indicated that impaired CA is associated with cerebral small vessel disease, cerebral hemorrhage, and unilateral middle cerebral artery stenosis [1–3]. These diseases are directly related to the cerebral artery. However, the circulatory system of the brain includes arteries and veins. Therefore, we aimed to explore the influence of cerebral venous system diseases on CA to enhance the understanding of CA and the pathophysiology of cerebral venous system diseases. Venous sinus stenosis (VSS) is a kind of cerebral venous system disease that obstructs venous blood outflow. Some studies have shown that it may cause increased intravenous pressure, decreased regional blood flow, destruction of the blood-brain barrier, and intracranial hypertension [4]. All of these changes may have an impact on artery function. Thus, VSS may be related to CA.

In this case, transcranial Doppler ultrasound and noninvasive continuous blood pressure measurements were used to obtain high temporal resolution blood flow velocity and blood pressure records. Dynamic cerebral autoregulation (dCA) was then calculated by transfer function analyses based on the response of blood flow to the transient changes in blood pressure [5].

## Case presentation

**Fig. 1:**
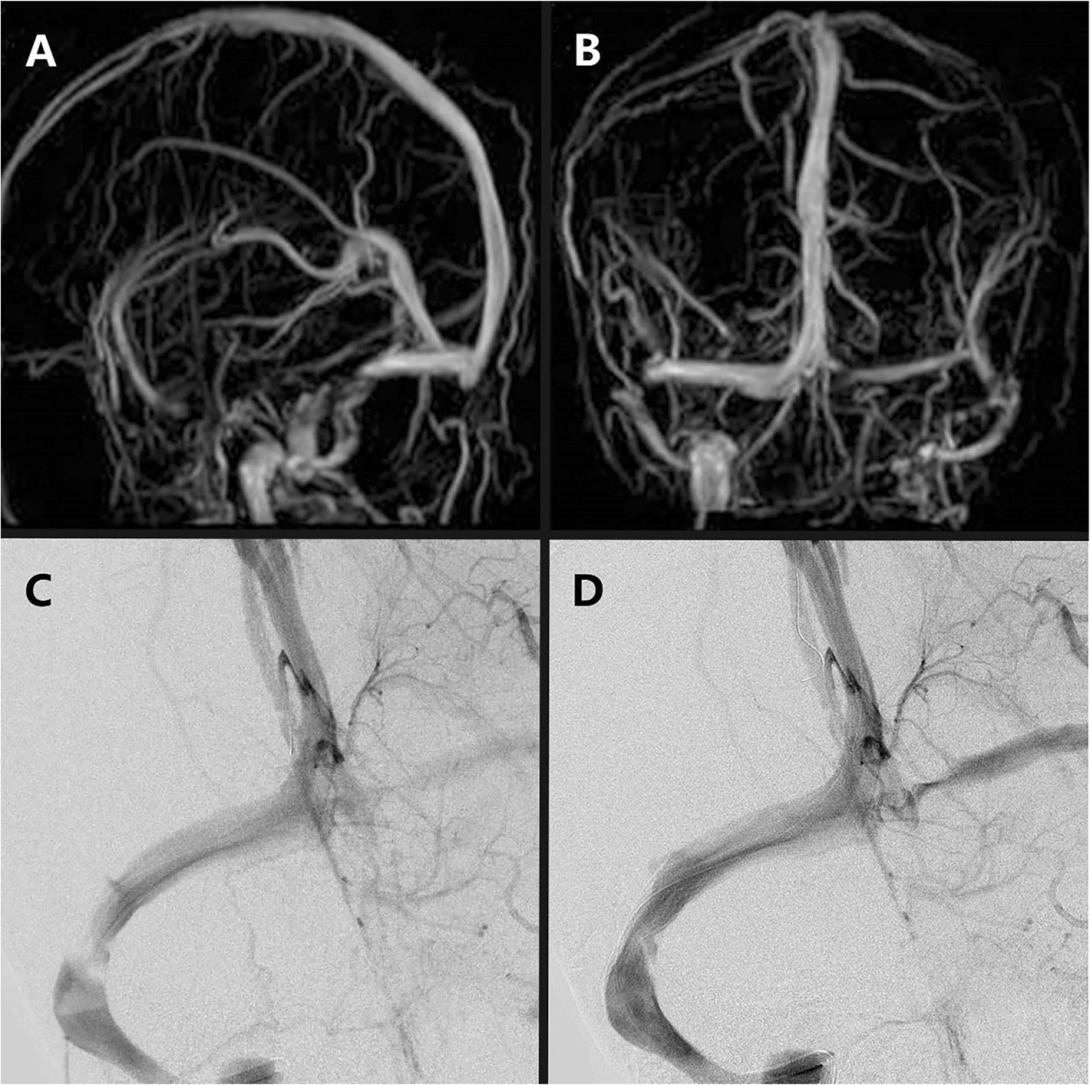
**a**, **b** Magnetic resonance venography suggests there is a stenosis at the border of the transverse sinus and sigmoid sinus on the right side. The picture shows that the patient’s left transverse sinus and sigmoid sinus are a congenital developmental disadvantage. **c** The digital subtraction angiography (DSA) clearly shows the stenosis at the border of the transverse sinus and sigmoid sinus on the right side. **d** After the patient accepted intracranial stenting, the stenting was placed at the border of the transverse sinus and sigmoid sinus on the right side. DSA shows the stent directly solved the VSS

A 50-year-old, right-handed woman who presented with transient blindness and headache from 2 years ago was admitted to the hospital. These symptoms worsened in the previous 2 months. A lumbar puncture showed that her intracranial pressure was more than 400 mmH_2_O. Magnetic resonance venography suggested stenosis at the border of the transverse sinus and sigmoid sinus on the right side (Fig. 1a and b). Digital subtraction angiography (DSA) showed stenosis at the border of the transverse sinus and sigmoid sinus on the right side and no sign of thrombus (Fig. 1c). The intravenous pressures were as follows: superior sagittal sinus, 48.9 cm H_2_O; torcular, 48.9 cm H_2_O; transverse sinus, 47.5 cmH_2_O; before stenosis, 47.5 cmH_2_O; and after stenosis, 12.2 cmH_2_O, the pressure gradient across the stenosis was 35.3 cmH_2_O. It can be concluded that the venous stenosis seriously hindered venous outflow. To relieve the VSS and reduce intracranial hypertension, treatment with venous sinus stent was recommended. The patient accepted intracranial stenting. The stenting was put at the border of the transverse sinus and sigmoid sinus on the right side (Fig. 1d), and venous sinus pressure was measured after the stenting: superior sagittal sinus, 31.2 cmH_2_O; torcular, 29.9 cmH_2_O; before stent, 29.9 cmH_2_O; at stent, 28.5 cmH_2_O; after stent, 25.8 cmH_2_O, the pressure gradient across the stenosis was 4.1 cmH_2_O; and near the sigmoid sinus, 23.1 cmH_2_O. Lumbar puncture after the operation showed the patient’s intracranial pressure to be 240 mmH_2_O. This indicates that, compared with the pressure before stenting, the stent directly solved the VSS.

**Fig. 2:**
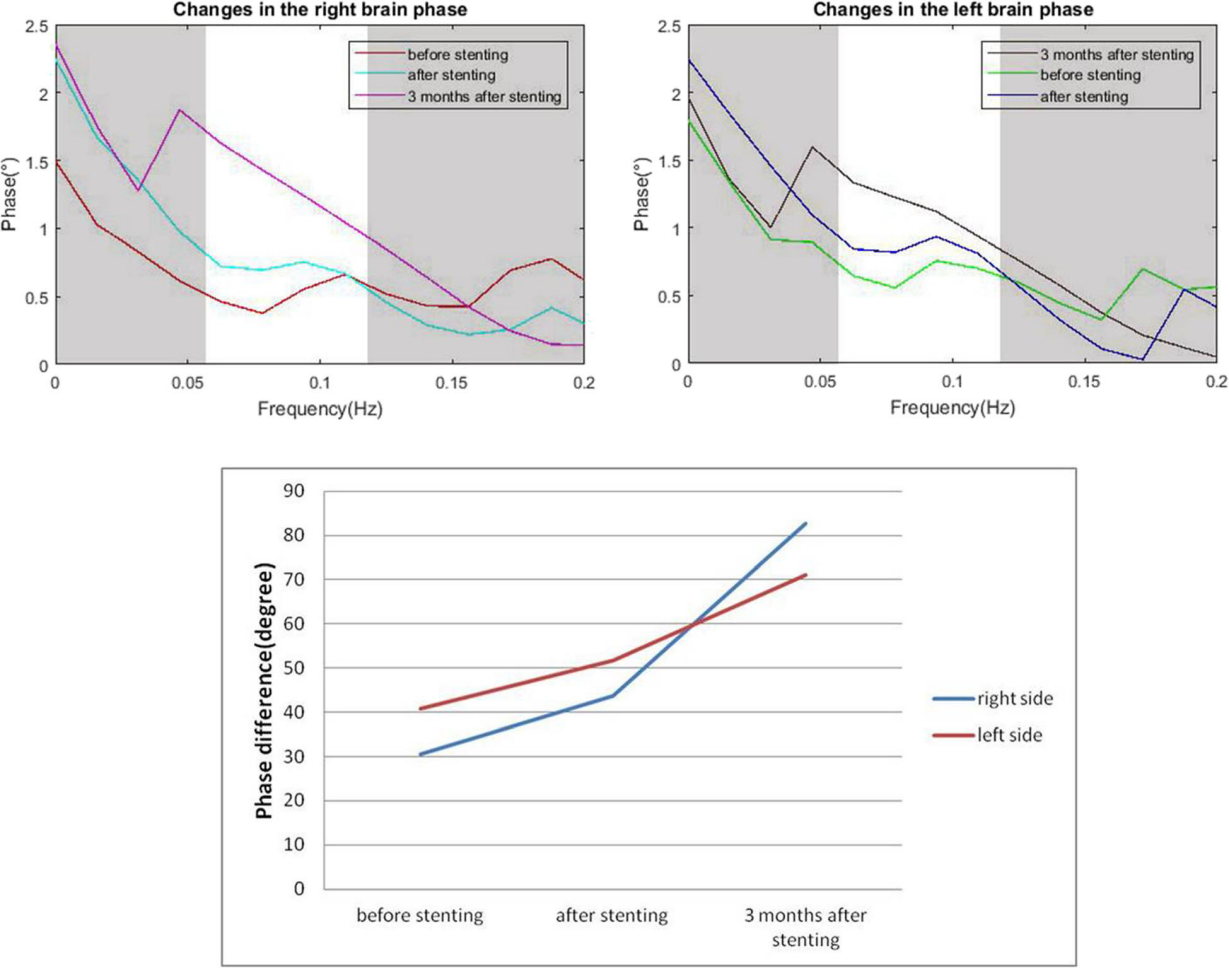
The autoregulatory parameter (phase difference) derived from the transfer function before stenting, after stenting, and 3 months later

In this case, transcranial Doppler ultrasound was used to measure high temporal resolution blood flow velocity. The relative low cost, ease of use, non-invasiveness, and excellent temporal resolution of transcranial Doppler ultrasound make it an ideal tool for examination of cerebrovascular function in both research and clinical settings. However, it cannot be used in patients with insufficient bilateral temporal bone windows for insonation of the middle cerebral artery. Measurements of dCA were performed at 3 time points: before stenting, after stenting, and 3 months later. The patient need to avoid alcohol and exercise 12 h before the measurement. And the measurements were made in a quiet, specialized laboratory with a constant temperature between 22 and 24 °C. Transcranial Doppler (EMS-9 PB, Delica, China) was used to evaluate cerebral blood flow velocity. The 2 MHz probe was fixed to a special head frame and detect blood flow velocity in the middle cerebral artery non-invasively at a depth of 45 to 60 mm. A servo-controlled plethysmograph (Finometer PRO, Netherlands) was used to record the natural arterial blood pressure of the right middle finger placed at the heart level. And a capnograph attached to a mask on a nasal tube was used to monitor end-tidal CO_2_. The 10-min monitoring data were processed by MATLAB software (MathWorks, Natick, MA, USA). The raw waveforms were sampled at 100 Hz for both arterial blood pressure and cerebral blood flow velocity. A cross-correlation function was used to achieve beat-to-beat alignment of data. To down sample the data to 1 Hz, a third-order Butterworth low-pass filter (cut-off at 0.5 Hz) was applied as an anti-alias filter. Dynamic cerebral autoregulation was evaluated using transfer function analysis [6, 7]. We used a transfer function to calculate the quotient of the cross-spectrum of arterial blood pressure and blood flow velocity in the middle cerebral artery signals and the autospectrum of arterial blood pressure in the frequency domain. In the frequency domain, we estimated the phase difference and gain within a low frequency range (0.06–0.12 Hz). Before venous sinus stenting, the dCA of the patient was as follows: gain (right side), 0.68 cm/s/mmHg; gain (left side), 0.68 cm/s/mmHg; phase difference (right side): 30.62 degrees; phase difference (left side), 40.74 degrees. End-tidal CO_2_: 37.1 mmHg. One day after the venous sinus stenting, the dCA of the patient was as follows: gain (right side), 0.74 cm/s/mmHg; gain (left side), 0.84 cm/s/mmHg; phase difference (right side), 43.77 degrees; phase difference (left side), 51.62 degrees. End-tidal CO_2_: 37.8 mmHg. The patient’s symptoms were significantly relieved. Three months after intracranial stenting, the dCA of the patient was as follows: gain (right side), 0.91 cm/s/mmHg; gain (left side), 0.76 cm/s/mmHg; phase difference (right side), 82.73 degrees; phase difference (left side), 71.20 degrees. End-tidal CO_2_ was 37.6 mmHg. Figure 2 shows the changes in the right- and left-brain phase difference. The patient reported no headache or blindness at the 3-month follow-up.

## Discussion and conclusion

VSS is a type of cerebral venous vascular disease. Several studies reported venous sinus stenting is a feasible treatment for refractory idiopathic intracranial hypertension and VSS [8–12]. Headache, papillary edema, and visual symptoms were improved in most of the patients after stenting [13–15]. We tried to relieve the symptoms of our patient by venous sinus stenting after strict screening. For this patient, the stenosis was at the border of her right transverse sinus and sigmoid sinus. The stenosis caused transient blindness and headache. After stenting, these symptoms were significantly relieved.

The underlying pathophysiology of cerebral vein occlusion remains controversial in clinical and experimental studies, in particular for the related hemodynamic and metabolic alterations [16, 17]. Currently, there has not been any study on dCA changes before and after venous sinus stenting. In this case, the cerebral blood flow regulation ability of the patient with VSS changed significantly before and after the stenosis was relieved; therefore, it can be supposed that the venous occlusion affects the artery function to some extent. VSS may affect the regulatory function of cerebral arteries in the following aspects: first, when the intracranial vein occludes, it can result in a decrease in cerebral blood flow. Some animal experiments have shown reduction of the regional cerebral blood flow after cerebral venous occlusion [18, 19]. Adequate perfusion of the affected brain tissue might still be possible at lower flow rates, if the blood is drained through collateral pathways [20]. Therefore, large areas of the brain can be functionally and metabolically disturbed but not irreversibly damaged after cerebrovenous occlusion [20], and obstruction of venous blood flow causes increased intravenous pressure, which reduces capillary perfusion pressure and decreases cerebral blood volume [4]. Second, increased venous and capillary pressure can lead to the destruction of the blood-brain barrier and the exudation of red blood cells [4, 21]. The function of the Na-K-ATP pump is affected by decreased cerebral blood flow and regional erythrocytopenia, as well as decreased energy supplied by cerebral arteries. These cause cellular edema and energy metabolism disorders, which in turn affect the function of cerebral arteries. Third, studies of animal models reported that in the normal cortex, 50 to 84% of all capillaries were perfused, and the remaining capillaries served as a reserve supply for possible pathological changes [4, 22–24]. Kurokawa et al. [25] demonstrated that the reserve capillaries are recruited, and the volume of the cerebral blood flow increases during the early phase of sinus occlusion. However, the reserve capillaries do not have good regulation ability, which has an effect on cerebral blood flow regulation. Finally, recent studies have demonstrated that intracranial hypertension was an independent cause of CA damage [26–28], and impaired CA may further aggravate the deterioration of intracranial hypertension [29, 30]. As the venous sinus is an important channel for cerebrospinal fluid outflow, any cause of VSS may lead to cerebrospinal fluid and blood outflow obstruction, resulting in intracranial hypertension. Furthermore, the increased intracranial pressure increases the external pressure of the venous sinus [31], and this further aggravates the VSS. This cycle leads to more severe intracranial hypertension. In this case, CA was significantly improved by intravenous sinus stenting, this result confirmed the association between intracranial pressure and CA.

Though several studies reported venous sinus stenting is a feasible treatment for refractory idiopathic intracranial hypertension and VSS [14, 15], there is no uniform standard for the indication of venous sinus stenting currently [14]. Furthermore, the long-term efficacy of intracranial sinus stenting has not been fully confirmed, and the treatment experience is limited [14]. This case provide a new way for us to judge the effectiveness of VSS treatment, however, future studies are recommended for determining the effectiveness of the treatment.

## Data Availability

The datasets used and/or analyzed during the current study are available from the corresponding author upon reasonable request.

## Abbreviations

CA: Cerebral autoregulation
dCA: Dynamic cerebral autoregulation
DSA: Digital subtraction angiography
VSS: Venous sinus stenosis
